# Dietary inflammation index is associated with dyslipidemia: evidence from national health and nutrition examination survey, 1999–2019

**DOI:** 10.1186/s12944-023-01914-z

**Published:** 2023-09-09

**Authors:** Xiaozhe Chen, Chunlei Hou, Lei Yao, Jianhua Li, Mingtai Gui, Mingzhu Wang, Xunjie Zhou, Bo Lu, Deyu Fu

**Affiliations:** Department of Cardiology, Yueyang Hospital of Integrated Traditional Chinese and Western Medicine, Shanghai University of Traditional Chinese Medicine, Shanghai, China

**Keywords:** Dyslipidemia, Dietary inflammation index, National Health and Nutrition Examination Survey, Cardiovascular Diseases

## Abstract

**Background and aims:**

This study aimed to investigate the association between the Dietary Inflammatory Index (DII) and dyslipidemia, as well as to evaluate the mortality risk associated with DII in participants with dyslipidemia.

**Methods:**

Data from the National Health and Nutrition Examination Survey database were divided into dyslipidemia and non-dyslipidemia groups. The association between DII and dyslipidemia was investigated using the weighted chi-square test, weighted t-test, and weighted logistic regression. Weighted Cox proportional hazards models were used to estimate the hazard ratios and 95% confidence intervals for all-cause and cardiovascular disease-related mortality within the dyslipidemia group.

**Results:**

A total of 17,820 participants, including 4,839 without and 12,981 with dyslipidemia were analyzed in this study. The results showed that DII was higher in the dyslipidemia group compared to the non-dyslipidemia group (1.42 ± 0.03 vs. 1.23 ± 0.04, *P* < 0.01). However, for energy, protein, carbohydrates, total fat, saturated fat, and iron, DII was lower in participants with dyslipidemia. Logistic regression analysis revealed a strong positive association between DII and dyslipidemia. The odds ratios for dyslipidemia from Q1 to Q4 were 1.00 (reference), 1.12 (0.96–1.31), 1.23 (1.04–1.44), and 1.33 (1.11–1.59), respectively. In participants with dyslipidemia, a high DII was associated with high all-cause and cardiovascular mortality.

**Conclusion:**

DII was closely associated with dyslipidemia. A pro-inflammatory diet may play a role in unfavorable consequences and is linked to both all-cause mortality and cardiovascular death in patients with dyslipidemia. Participants with dyslipidemia should pay attention to their anti-inflammatory dietary patterns.

**Supplementary Information:**

The online version contains supplementary material available at 10.1186/s12944-023-01914-z.

## Introduction

Dyslipidemia comprises a range of conditions primarily defined by elevations in lipoprotein cholesterol, including low-density lipoprotein (LDL) and non-high-density lipoprotein cholesterol, as well as elevated triglycerides (TG) [1]. In the United States, 29% and 26% of adults have elevated LDL and TG levels, respectively [2]. Early accumulation of total cholesterol (TC) increases the risk of cardiovascular diseases (CVDs) by 2–3 times [3], which is a high-risk factor for stroke and death [4]. According to the U.S. Preventive Services Task Force, CVDs account for > 25% of all deaths in the United States [5]. Dyslipidemia causes a huge health and economic burden worldwide, and medical treatment has long been committed to improving dyslipidemia [6]; however, there is a serious lack of awareness of the etiology and prevention of this disease [7].

Dyslipidemia is primarily caused by hereditary, nutritional, and systemic diseases [8, 9]. Anti-inflammatory dietary patterns (such as the Mediterranean diet [10], dash diet [11], and Nordic diet [12]) are considered to have preventive effects. Potential mechanisms include improving insulin resistance [13], altering the gut microbiome [14], reducing mucosal and systemic inflammatory responses [15], and affecting epigenetic links such as DNA methylation and acetylation [16]. Obesity is another important risk factor for dyslipidemia, which has been recognized as chronic low-grade inflammation; a high-sugar, high-fat diet leads to increased harmful metabolites and systemic inflammation, which ultimately promotes the progression of obesity [17, 18].

The Dietary Inflammatory Index (DII) is a tool used to assess the level of an individual’s dietary inflammation by scoring the pro- or anti-inflammatory levels of various diets, which helps to clarify the relationship between diet-related inflammation and various metabolic diseases [19].

Increasing attention has been paid to the important role of systemic chronic inflammation in the occurrence and development of obesity [17], metabolic syndrome [20], cardiovascular metabolic diseases [21], diabetes mellitus (DM) [22], tumors [23] and other major chronic noncommunicable diseases [24] that threaten human health. Most studies were clinical trials, limited by the number of participants and short follow-up periods. No study has analyzed the association between DII and dyslipidemia and the risk on mortality outcomes in participants with dyslipidemia in large, well followed-up public databases so far.

In this study, we compared DII between individuals with and without dyslipidemia, explored the dose relationship between DII and dyslipidemia, and investigated the association between DII and all-cause mortality and CVDs mortality in the dyslipidemia group based on the National Health and Nutrition Examination Survey (NHANES) databases.

## Methods

### Data extraction

NHANES is a nationwide cross-sectional survey conducted by the National Center for Health Statistics in the United States. Detailed information on the design of the continuous NHANES is available at http://www.cdc.gov/nchs/nhanes/index.htm. All study protocols were approved by the Ethics Review Board of the National Center for Health Statistics. Ethical accreditation of our study was provided by the NHANES institutional review board https://www.cdc.gov/nchs/nhanes/irba98.html (Protocol #98 − 12, Protocol #2005-06, Protocol #2011-17, Protocol #2018-01).

Data from 1999 to 2019 were gathered from the NHANES database [25]. The program uses the United States Department of Agriculture’s automated multiple-pass method (AMPM) to collect dietary information from representative participants. Participants were selected based on a national sampling design. The AMPM approach is a research-based multiple-pass approach aimed at minimizing interviewer recall regarding food consumption [26]. Five steps were designed to enhance the completeness and accuracy of food recall. Additionally, at the conclusion of the interview, the participants were asked to indicate whether their food intake on the recalled day was greater, similar, or significantly lower than their usual consumption [27]. Finally, the nutrient profiles of every food and beverage were calculated using the Food Nutrient Database for Dietary Studies.

**Fig. 1:**
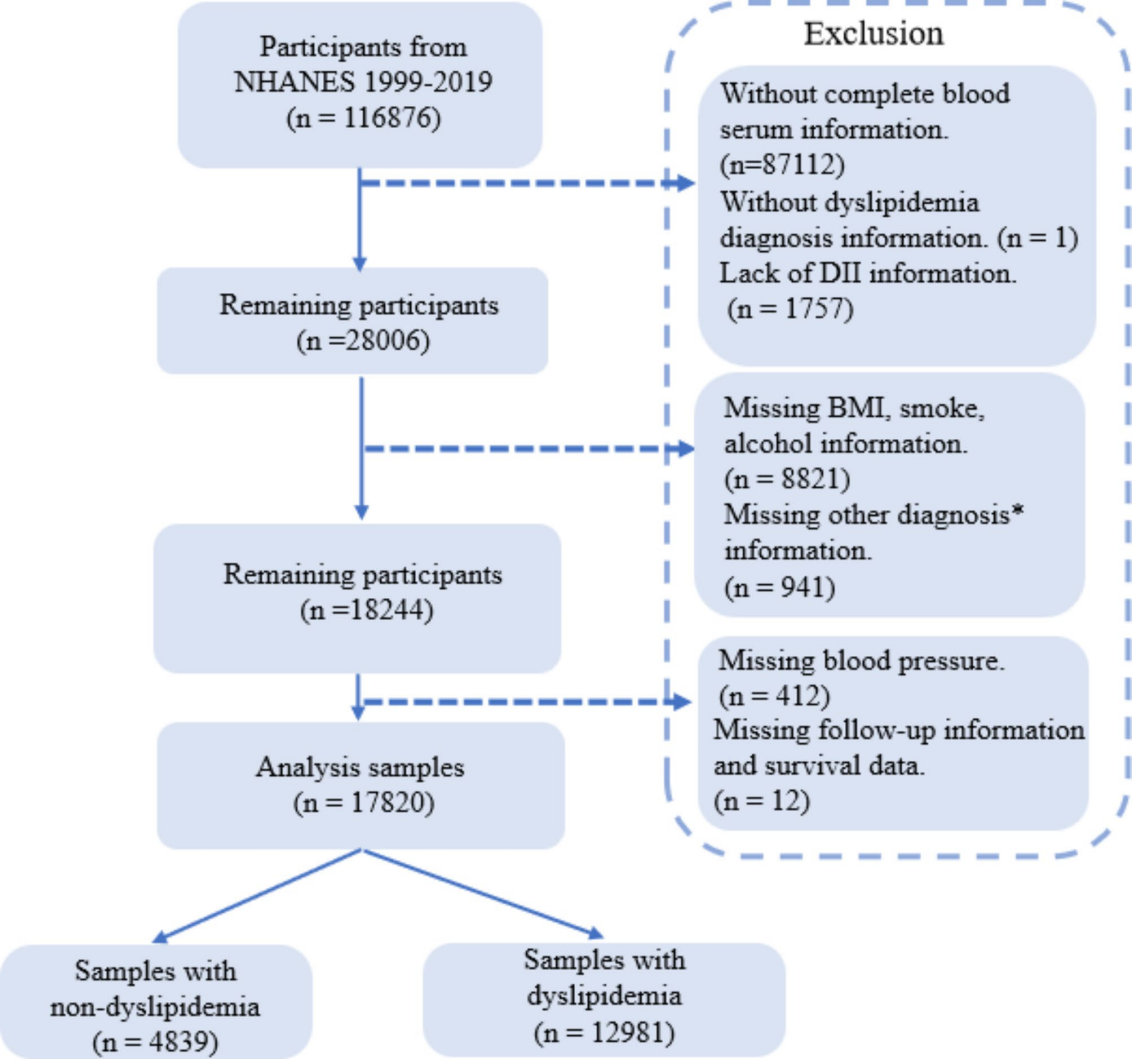
Flow chart of the screening process for the selection of participants in NHANES (1999–2019) Note: *diagnosis including: hypertension, DM, CAD, CHF, stroke and cancer

The inclusion criteria were: (1) participants at least 20 years old; (2) complete and accessible participants information; (3) complete mortality follow-up information; and (4) complete information regarding all exposure variables, outcome variables, and covariables. The collection is detailed in the flow chart (Fig. 1). Overall, 116,876 participants were included. After excluding participants without serum information (serum lipids and routine blood tests), dyslipidemia diagnosis information, DII information, and information about other covariables, including body mass index (BMI), smoking status, alcohol status, and other related diseases (hypertension, diabetes mellitus [DM], coronary artery disease [CAD], chronic heart failure [CHF], stroke, and cancer), 17,820 participants were ultimately included in our analysis.

### Exposure variable

DII was set as the exposure variable. It was calculated by adding the scores for each dietary component consumed within 24 h. We calculated DII according to the protocol described by Shivappa et al. [19]. The calculation of DII was based on a world database containing dietary intake data, as described by Shivappa et al. Robust estimates of the mean and standard deviation for each parameter were provided. The centered percentile value for each parameter was multiplied by the overall food parameter-specific inflammatory effect score. Food parameter-specific DII scores were summed to obtain the overall DII score for each individual. DII based on less than 30 food parameters reportedly maintains its predictive capacity. In the NHANES database, 28 food parameters are available, including energy, protein, carbohydrate, fiber, total fat, saturated fat, monounsaturated fatty acids (MUFA), polyunsaturated fatty acids (PUFA), cholesterol, Vitamin A, β-carotene, thiamin, riboflavin, niacin, Vitamin B6, folic acid, Vitamin B12, Vitamin C, Vitamin D, Vitamin E, magnesium, iron, zinc, selenium, caffeine, alcohol, and N3 and N6 fatty acids. By summarizing each DII score, we obtained an “overall DII score,” which is the final DII score for each topic that defines anti-and proinflammatory diets. DII is lower with an anti-inflammatory diet and higher with a pro-inflammatory one.

### Outcome variable

The main outcomes were dyslipidemia status and mortality. Dyslipidemia was defined as an elevated TG (≥ 150 mg/dL) TC level (≥ 200 mg/dL or 5.18 mmol/L, LDL cholesterol ≥ 130 mg/dL or 3.37 mmol/L, or high-density lipoprotein [HDL] cholesterol < 40 mg/dL or 1.04 mmol/L in men and < 50 mg/dL or 1.30 mmol/L in women) or intake of cholesterol-lowering agents [28].

To investigate whether DII is associated with the risk of all-cause and CVD-related mortality in patients with dyslipidemia, we performed a survival analysis in the dyslipidemia group. Mortality status was determined by cross-referencing the NHANES data with the national death index records, commencing from the baseline survey date until December 31, 2019. The identification of fatalities resulting from CVDs (specifically I20–I51, I11, I13, I00–I09) was based on the 10th revision of the International Statistical Classification of Diseases and Related Health Problems (ICD-10).

### Covariable

We included covariables associated with DII and dyslipidemia based on previous studies [29, 30]. The variables included age, sex, race, education, annual family income, BMI, C-reactive protein (CRP), alcohol consumption, smoking status, hypertension, DM, CAD, CHF, stroke, and cancer. The following covariables were extracted directly from the NHANES database according to participants’ self-reported data: age, sex, and race.

BMI was calculated as weight (kg) divided by height squared (m^2^) [31].

Hypertension was diagnosed when one of the following conditions was met [32]: (1) participant responded affirmatively to the inquiry “Have you received a definitive diagnosis of hypertension?”; (2) participant displayed a systolic blood pressure (SBP) measurement ≥ 140 mmHg and/or a diastolic blood pressure (DBP) measurement ≥ 90 mmHg; or (3) current use of antihypertensive medication.

DM was diagnosed when any of the following conditions were met [33]: (1) participant answered affirmatively to the question “Do you have a clear diagnosis of DM?”; (2) glycohemoglobin levels > 6.5%; (3) fasting plasma glucose levels ≥ 7.0 mmol/L; (4) randomly assigned blood glucose levels ≥ 11.1 mmol/L; (5) 2-hour oral glucose tolerance test levels ≥ 11.1 mmol/L; or (6) the participant was taking medications for diabetes or insulin.

CHF, CAD, cancer, and stroke were diagnosed according to the participants’ answers to the question, “Do you have a clear diagnosis of CHF/CAD/cancer/stroke?”

Education level was classified as “below high school,” “high school,” and “above high school.”

Alcohol intake was categorized as never (< 12 drinks in lifetime), former drinker (≥ 12 drinks in 1 year and consumed none in the past year, or had not consumed alcohol in the past year but drank ≥ 12 drinks in their lifetime), current mild drinker (≤ 1 drink per day for women or ≤ 2 drinks per day for men on average over the past year), current moderate drinker (≤ 2 drink per day for women or ≤ 3 drinks per day for men on average over the past year), or current heavy drinkers (> 3 drinks per day for women or > 4 drinks per day for men on average over the past year) [34].

### Statistical analysis

In our study, wtdr4 year and wtdrd1 were used as weighted variables. Wtdr4 year was the sample weighting code for day-1 dietary in 1999–2002 while wtdrd1 were the sample weighting code for day-1 dietary in 2003–2019. The detail information about sample weighting code could be found on https://wwwn.cdc.gov/nchs/nhanes/search/default.aspx.

Our study conducted two sets of quartile calculations: one based on DII of all participants and the other based on the dyslipidemia subgroup. The former were labeled as Q1, Q2, Q3, and Q4, while the latter were labeled as q1, q2, q3, and q4. Three adjustment variables of the models were used for the dose-related and survival analyses. Model 1 was adjusted for age and sex; Model 2 was adjusted for age, sex, race, education, and BMI; and Model 3 was adjusted for age, sex, race, education, BMI, smoking, alcohol consumption, and comorbidities, including hypertension, DM, CAD, CHF, stroke, and cancer. All data were analyzed using R (version 4.2.3; R Foundation for Statistical Computing, Vienna, Austria) and R Studio. *P* < 0.05 was considered as a statistically significant. For continuous variables, the mean and standard error (SE) were used, and weighted t-tests were used for comparison. For categorical variables, we used the number of cases and weighted prevalence for description and a chi-squared test for comparison between groups.

Univariate logistic regression analyses were performed to investigate the association between dyslipidemia and DII. A multivariate logistic regression model was then used to analyze the relationship between DII and dyslipidemia after adjusting for covariables. The adjusted variables included sex, BMI, race, family income, education, comorbidities (CHF, DM, CAD, hypertension, cancer, and stroke), and smoking, and alcohol status. Dose-related analyses of DII in participants with dyslipidemia were performed using multivariate logistic regression. The outcome variable was dyslipidemia, and the control group was participants without dyslipidemia. We calculated *P*_for trend_ to assess the association between DII and dyslipidemia, adjusted with the three aforementioned models. We conducted a subgroup analysis of age, sex, race, family income, education level, BMI, comorbidities, smoking, and alcohol consumption.

Finally, a survival analysis was performed in the subgroup of participants with dyslipidemia to reveal the association between the DII and the risk of all-cause and CVD-related mortality in these participants. Cox regression analysis was performed using DIIs of participants with dyslipidemia. The Cox proportional hazards model was used to estimate the hazard ratios (HRs) and 95% confidence intervals (CIs) for the association between concentrations and all-cause and cause-specific mortality.

## Results

### Baseline characteristics of participants

Overall, 17,820 participants were involved, representing a population of 117,825,860 US citizens with robust follow-up data. Table 1 shows the basic information of participants with and without dyslipidemia. Participants with dyslipidemia (12,981) accounted for approximately 72.84% of all the participants in our study. Participants with dyslipidemia exhibited higher DII (1.42 ± 0.03 vs. 1.23 ± 0.04), BMI (29.15 ± 0.11 vs. 26.24 ± 0.12 kg/m^2^), and blood pressure (SBP: 123.57 ± 0.27 mmHg vs. 117.51 ± 0.35 mmHg; DBP: 71.68 ± 0.22 mmHg vs. 69.42 ± 0.26 mmHg). Additionally, participants with dyslipidemia were older (50.13 ± 0.28 years vs. 40.21 ± 0.36 years), with a higher CRP (0.44 ± 0.02 mg/L vs. 0.31 ± 0.01 mg/L) and likelihood for comorbidities (hypertension, DM, CAD, CHF, stroke, and cancer). The baseline characteristics of the participants across DII quartiles are shown in Supplementary Table S1. Compared with the Q1, Q2, and Q3 groups, the Q4 group had the highest proportion of participants with dyslipidemia. The results also suggested a gradual decrease in the proportion of participants without dyslipidemia in the Q1–Q4 groups, which were 30.04%, 26.57%, 25.38%, and 23.74%, respectively. In Q4, the proportion of participants with dyslipidemia reached 76.26%, accompanied by a higher BMI of 29.10 ± 0.16 kg/m^2^. Furthermore, the Q4 group exhibited a higher incidence of comorbidities, including CHF, DM, stroke, and hypertension than the Q1–Q3 groups. Moreover, Q4 showed the highest CRP levels (0.52 ± 0.03 mg/L).

**Table 1:** Baseline characteristics of participants (n = 17,820) by dyslipidemia status

Variable	Total	Non- Dyslipidemia	Dyslipidemia	*P* value
	n = 17,820^a^	n = 4839 ^a^	n = 12,981^a^	
Age	47.49 ± 0.26	40.21 ± 0.36	50.13 ± 0.28	< 0.01
Sex (%)				0.09
Female	8880(49.83)	2314(49.00)	6566(51.37)	
Male	8940(50.17)	2525(51.00)	6415(48.63)	
Race (%)				< 0.01
Mexican American	3081(17.29)	757 (7.27)	2324(7.04)	
Non-Hispanic Black	3459(19.41)	1180(13.18)	2279(8.81)	
Non-Hispanic White	8463(47.49)	2103(68.74)	6360(74.08)	
Other Hispanic	1420(7.97)	348(4.86)	1072(5.07)	
Other Race	1397(7.84)	451(5.95)	946(4.99)	
Annual family income (%)				0.35
<$20,000	4501(25.26)	1206(19.36)	3295(20.24)	
≥$20,000	13,319(74.74)	3633(80.64)	9686(79.76)	
Education (%)				< 0.01
< High school	1955(10.97)	396(4.69)	1559(6.06)	
> High school	9178(51.5)	2751(62.32)	6427(56.54)	
High school	6687(37.53)	1692(32.99)	4995(37.40)	
TG (mmol/L)	1.40 ± 0.01	0.88 ± 0.01	1.59 ± 0.01	< 0.01
TC (mmol/L)	5.07 ± 0.01	4.38 ± 0.01	5.33 ± 0.01	< 0.01
HDL cholesterol (mmol/L)	1.38 ± 0.01	1.53 ± 0.01	1.32 ± 0.01	< 0.01
LDL cholesterol (mmol/L)	3.06 ± 0.01	2.45 ± 0.01	3.28 ± 0.01	< 0.01
CRP (mg/dl)	0.41 ± 0.01	0.31 ± 0.01	0.44 ± 0.02	< 0.01
BMI (kg/m^2^)	28.38 ± 0.09	26.24 ± 0.12	29.15 ± 0.11	< 0.01
SBP (mmHg)	121.96 ± 0.23	117.51 ± 0.35	123.57 ± 0.27	< 0.01
DBP (mmHg)	71.08 ± 0.19	69.42 ± 0.26	71.68 ± 0.22	< 0.01
Hypertension (%)	7568(42.47)	1332(22.77)	6236(42.86)	< 0.01
DM (%)	5975(33.53)	947(13.88)	5028(29.18)	< 0.01
CAD (%)	735(4.12)	63(1.07)	672(4.50)	< 0.01
CHF (%)	528(2.96)	60(0.74)	468(2.88)	< 0.01
Stroke (%)	640(3.59)	83(1.56)	557(3.06)	< 0.01
Cancer (%)	1646(9.24)	296(6.10)	1350(10.55)	< 0.01
Smoke (%)				< 0.01
Former	4579(25.7)	1010(22.88)	3569(27.53)	
Never	9507(53.35)	2792(54.54)	6715(50.63)	
Now	3734(20.95)	1037(22.58)	2697(21.84)	
Alcohol (%)				< 0.01
Former	3104(17.42)	606(11.85)	2498(16.23)	
Heavy	3531(19.81)	1141(23.99)	2390(18.53)	
Mild	6131(34.41)	1598(34.25)	4533(38.31)	
Moderate	2673(15)	860(19.85)	1813(15.75)	
Never	2381(13.36)	634(10.06)	1747(11.17)	
DII	1.37 ± 0.03	1.23 ± 0.04	1.42 ± 0.03	< 0.01

Note: Mean ± SE for continuous variables; *P* value was calculated by weighted t test, Number (%) for categorical variables: The *P* value was calculated by weighted chi-square test. ^a^Unweighted number of observations in dataset

### Comparison of DII and component of DII between the dyslipidemia and non-dyslipidemia groups

Compared to participants without dyslipidemia, those with dyslipidemia had higher scores for fiber, MUFA, PUFA, vitamins A/E/B6, thiamin, riboflavin, niacin, folic acid, magnesium, zinc, selenium, alcohol, and N-6 fatty acids. However, they had lower energy, protein, carbohydrate, total fat, saturated fat, and iron scores (Supplementary table S2).

### Association between DII and dyslipidemia

**Fig. 2:**
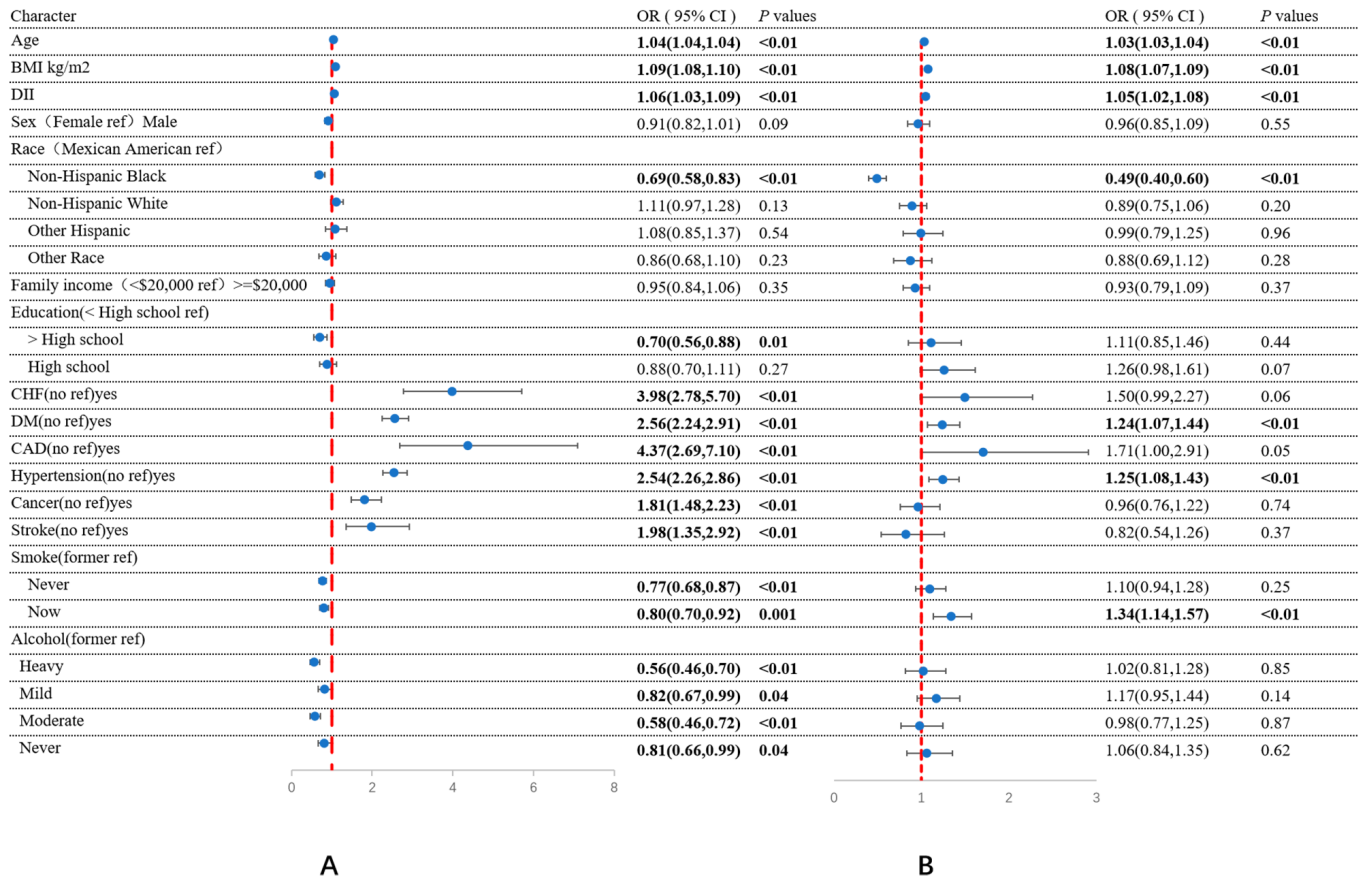
Logistic regression of factors associated with dyslipidemia Note: DII was associated with dyslipidemia in both weighted univariable logistic regression (A) and weighted multivariable logistic regression (B). The *P* value was calculated by weighted univariable logistic regression and multivariable logistic regression. Covariable including age, BMI, sex, race, family income, education, hypertension, DM, CAD, CHF, stroke, and cancer, smoking and alcohol intake

The univariate logistic regression analysis is presented in Fig. 2A. DII was strongly and positively associated with dyslipidemia. The odds ratio (OR) (95% confidence interval [CI]) was estimated to be 1.06 (1.03–1.09), indicating that the odds of having dyslipidemia increased by 1.06 times for every one-unit increase in DII (*P* < 0.01). Age, BMI, comorbidities (CHF, DM, cancer, stroke, CAD, and hypertension), smoking status, and alcohol intake were also associated with dyslipidemia. In multivariate logistic regression analysis, after adjusting for sex, BMI, race, family income, education, comorbidities (hypertension, DM, CAD, CHF, stroke, and cancer), and smoking, and alcohol status (Fig. 2B), DII was still associated with dyslipidemia. The OR (95% CI) was 1.05 (1.02–1.08), indicating that the odds of having dyslipidemia increased by 5% for every one-unit increase in DII (*P* < 0.01).

### Dose-response analysis of DII and dyslipidemia

Multiple regression analysis was conducted across the DII quartiles to further explore the dose-response relationship between dyslipidemia and DII (Table 2). After adjusting for smoking, alcohol and comorbidities (hypertension, DM, CAD, CHF, stroke, and cancer), the OR and 95% CI from Q1 to Q4 were 1.00 (reference), 1.12 (0.96–1.31), 1.23 (1.04–1.44), and 1.33 (1.11–1.59), respectively (*P*_for trend_ <0.01), indicating a persistent dose-response relationship between DII and dyslipidemia.

**Table 2:** Odds ratios (95% confidence intervals) of dyslipidemia across quartiles of DII regression

	Model1		Model2		Model3	
Character	OR (95% CI)	*P* value	OR (95% CI)	*P* value	OR (95% CI)	*P* value
Q1(-4.67,0.26]	ref	ref	ref	ref	ref	ref
Q2(0.26,1.76]	1.19(1.02,1.38)	0.02	1.14(0.98,1.33)	0.09	1.12(0.96,1.31)	0.13
Q3(1.76,2.96]	1.32(1.13,1.54)	< 0.01	1.25(1.07,1.46)	0.01	1.23(1.04,1.44)	0.01
Q4(2.96,5.50]	1.52(1.31,1.76)	< 0.01	1.39(1.17,1.64)	< 0.01	1.33(1.11,1.59)	< 0.01
*P*_for trend_		< 0.01		< 0.01		< 0.01

Note: ref: reference; Model 1 adjusted for age and sex; Model 2 adjusted for age, sex, race, education and BMI; Model 3 adjusted for age, sex, race, education, BMI, smoking, alcohol and comorbidities (hypertension, DM, CAD, CHF, stroke, and cancer)

### Subgroup analysis

Significant moderating effects were found in the subgroup analysis stratified by family income (*P*_for interaction_ = 0.03). A stronger association was observed in participants with an annual family income <$20,000 than in those with annual family income >$20 000; ORs (95% CI) were 1.13 (1.07–1.20) and 1.04 (1.01–1.08), respectively. However, DII was still associated with dyslipidemia in participants with a household income >$20,000 (*P*_for trend_ = 0.01). The association between DII and dyslipidemia was more significant in participants without comorbidities (CHF, DM, stroke, CAD, cancer) (*P*_for trend_ <0.05). There was no significant interaction between the subgroups (*P*_for interaction_ >0.05) (Table 3).

**Table 3:** Subgroup analysis of association between DII and dyslipidemia among US in NHANES 1999–2019

Subgroups	OR(95% CI)	*P*_for trend_	*P*_for interaction_
Age			0.19
> 60	1.10(1.04,1.18)	0.002	
40–60	1.03(0.98,1.09)	0.248	
20–39	1.09(1.04,1.14)	< 0.01	
Sex			0.12
Male	1.03(0.99,1.07)	0.10	
Female	1.08(1.04,1.13)	< 0.01	
Race			0.73
Non-Hispanic White	1.07(1.03,1.10)	< 0.01	
Non-Hispanic Black	1.09(1.02,1.17)	0.01	
Other Hispanic	1.10(0.97,1.24)	0.12	
Mexican American	1.06(1.00,1.13)	0.06	
Other Race	1.01(0.92,1.12)	0.77	
Family income			0.03
≥$20,000	1.04(1.01,1.08)	0.01	
<$20,000	1.13(1.07,1.20)	< 0.01	
Education			0.34
> High school	1.04(1.00,1.07)	0.04	
High school	1.08(1.02,1.14)	< 0.01	
< High school	1.04(0.95,1.15)	0.39	
BMI			0.34
20–25	1.06(1.01,1.11)	0.02	
25–30	1.04(0.98,1.11)	0.14	
> 30	0.99(0.94,1.05)	0.79	
<=20	1.09(0.97,1.23)	0.13	
CHF			0.63
No	1.06(1.03,1.09)	< 0.01	
Yes	1.11(0.91,1.35)	0.30	
DM			0.96
No	1.05(1.02,1.08)	< 0.01	
Yes	1.05(0.99,1.12)	0.12	
Stroke			0.60
No	1.06(1.03,1.09)	< 0.01	
Yes	1.01(0.84,1.21)	0.93	
CAD			0.71
No	1.06(1.03,1.09)	< 0.01	
Yes	1.03(0.85,1.24)	0.79	
Hypertension			0.92
No	1.05(1.02,1.09)	< 0.01	
Yes	1.05(1.00,1.10)	0.04	
Cancer			0.84
No	1.06(1.03,1.09)	< 0.01	
Yes	1.05(0.95,1.16)	0.31	
Smoke			0.24
Never	1.05(1.01,1.09)	0.02	
Former	1.07(1.02,1.13)	0.01	
Now	1.11(1.05,1.17)	< 0.01	
Alcohol			0.13
Mild	1.05(1.00,1.10)	0.06	
Moderate	1.11(1.05,1.18)	< 0.01	
Former	1.10(1.03,1.18)	0.01	
Heavy	1.01(0.95,1.07)	0.79	
Never	1.10(1.02,1.19)	0.02	

### Survival analysis for DII with all-cause CVD-related mortality in participants with dyslipidemia

In the cohort of 12,981 participants with dyslipidemia, 2,203 all-cause deaths were observed during the follow-up period, of which 588 were related to CVDs. After adjusting for age, sex, race, education, BMI, smoking, alcohol consumption, and comorbidities (CHF, DM, stroke, CAD, hypertension, and cancer) in Model 3, DII remained highly associated with all-cause CVD-related mortality in patients with dyslipidemia. The multivariate-adjusted HRs (95% CI) for all-cause mortality from Q1 to Q4 were 1.00 (reference), 1.39 (1.20–1.62), 1.37 (1.13–1.65), 1.50 (1.24–1.82), respectively (*P* < 0.01). For CVD-related mortality, HRs from Q1 to Q4 were 1.00 (reference), 1.46 (1.00–2.13), 1.38 (0.94–2.01), 2.07 (1.38–3.09), respectively (*P* < 0.01) (Table 4).

**Table 4:** All-cause mortality and CVD mortality in dyslipidemia participants

	Model1		Model2		Model3		Deaths/Total
All-cause mortality in dyslipidemia across quartiles of DII							2203/12,981
Character	HR (95% CI)	*P* value	HR (95% CI)	*P* value	HR (95% CI)	*P* value	
q1 [-4.63,0.35]	ref	ref	ref	ref	ref	ref	431/3248
q2 (0.35,1.82]	1.52(1.30,1.76)	< 0.01	1.48(1.26,1.73)	< 0.01	1.39(1.20,1.62)	< 0.01	572/3243
q3 (1.82,2.99]	1.64(1.35,1.99)	< 0.01	1.54(1.27,1.88)	< 0.01	1.37(1.13,1.65)	< 0.01	607/3245
q4 [2.99,5.47]	1.93(1.57,2.37)	< 0.01	1.81(1.49,2.20)	< 0.01	1.50(1.24,1.82)	< 0.01	593/3345
P for trend		< 0.01		< 0.01		< 0.01	
CVD mortality in dyslipidemia across quartiles of DII							558/12,981
q1 [-4.63,0.35]	ref	ref	ref	ref	ref	ref	113/3248
q2 (0.35,1.82]	1.60(1.08,2.35)	0.02	1.53(1.05,2.23)	0.03	1.46(1.00,2.13)	0.05	147/3243
q3 (1.82,2.99]	1.72(1.19,2.48)	< 0.01	1.57(1.10,2.25)	0.01	1.38(0.94,2.01)	0.10	161/3245
q4 [2.99,5.47]	2.72(1.83,4.05)	< 0.01	2.52(1.71,3.73)	< 0.01	2.07(1.38,3.09)	< 0.01	167/3345
*P*_for trend_		< 0.01		< 0.01		< 0.01	

Note: ref: reference; Model 1 adjusted for age and sex; Model 2 adjusted for age, sex, race, education and BMI; Model 3 adjusted for age, sex, race, education, BMI, smoking, alcohol and comorbidities (hypertension, DM, CAD, CHF, stroke, and cancer)

## Discussion

This study represented an initial analysis of the association between DII and dyslipidemia based on the NHANES database. These findings indicate that a proinflammatory diet is associated with dyslipidemia and that a dose-response relationship exists between DII and dyslipidemia. Additionally, survival analysis suggested that dyslipidemia with a higher DII score was positively associated with a high risk of all-cause CVD-related mortality.

### Comparison with other studies

DII has been used to evaluate the inflammatory propensity for individual dietary intake. This was first proposed by Hébert et al. (2009) [35]. Tyrovolas et al. reported the relationship between DII and CVD risk factors (assessed as obesity, DM, hypertension, and hypercholesterolemia) and suggested that the CVD risk factors among participants on a proinflammatory diet were 1.37–1.50 times higher compared to those on an anti-inflammatory diet [36]. In another clinical study, DII was found to increase waist circumference and TG levels in overweight and obese women [37]. However, most of these studies were clinical studies and were limited by their small sample sizes and short follow-up periods. Hence, we investigated the association between DII and dyslipidemia based on NHANES data. Our results suggested a strong relationship between a proinflammatory diet and dyslipidemia. Subgroup analyses suggested that DII was more strongly associated with dyslipidemia in households with annual incomes <$20 000. A proinflammatory diet was also strongly associated with all-cause and cardiovascular mortality in participants with dyslipidemia.

### Possible mechanisms between Dietary Inflammatory and Dyslipidemia

Our study found that DII for fiber, MUFA, PUFA, vitamin A/E, vitamin B (including B6, thiamin, riboflavin, niacin, and folic acid), magnesium, and zinc in participants with dyslipidemia were significantly higher than those in participants without dyslipidemia. Possible mechanisms are as follows.

Fiber: Previous research has demonstrated that a higher fiber content can potentially mediate the beneficial effects of risk markers associated with CVDs [38]. These effects are attributed to various mechanisms such as enhanced insulin sensitivity, decreased oxidative stress, and cytokine production [39]. Additionally, it has been found that fiber exerts anti-inflammatory properties [40].

MUFA and PUFA: Studies have found that MUFA can mitigate inflammation by modulating the production of inflammatory mediators and regulating neutrophil infiltration [41]. Potential underlying mechanisms include the reduction of endoplasmic reticulum stress [42], enhancement of vascular endothelial inflammation, and systemic inflammatory responses [43].

Vitamins A and E: Fat-soluble vitamins possess antioxidant properties [44] and can interact with lipoproteins. Their action is linked to the production of chylomicrons in the gut, which subsequently affect lipid absorption and metabolism [45, 46].

Folic acid and Vitamin B6 are essential nutrients for nucleic acid synthesis and methyl generation [47]. Vitamin B6, in particular, has been found to reduce endogenous cholesterol and lipid synthesis, while enhancing cholesterol transport to liver cells [48].

Niacin: Niacin has been extensively studied for its ability to regulate fat metabolism. It can modulate the expression of lipoproteins [49, 50], diacylglycerol acyltransferase [51], and genes related to fat metabolism [52, 53]. Niacin promotes endothelium-dependent vasodilation and reduces vascular inflammation [54].

Thiamin: Thiamin, in its bioactive form (thiamine pyrophosphate), plays a crucial role in maintaining the balance of oxidative metabolism in the body. It regulates glucose and lipid metabolism by influencing lipid peroxidation product levels and glutathione reductase activity [55].

Riboflavin: Riboflavin affects the activity of lipid-metabolizing enzymes, such as 3-hydroxy-3-methyl glutaryl coenzyme A reductase and lecithin-cholesterol acyltransferase, thereby regulating blood lipid levels [56, 57]. Additionally, studies have shown a negative association between riboflavin and the intestinal flora, suggesting a potential mechanism for regulating lipid metabolism [58].

Magnesium: Increasing magnesium intake improves chronic metabolic disorders and cardiovascular diseases by reducing low-grade inflammation [59]. This effect is believed to be mediated by the regulation of reactive oxygen species activity [60]. Furthermore, magnesium can regulate enzymes involved in liver lipid metabolism, thereby reducing blood lipid levels [60, 61].

### Implication for the clinical practice

Changes in the distribution of large amounts of nutrients (such as fats), low-carbohydrate diets, and caloric restriction appear to be effective strategies for ameliorating inflammation and reducing serum lipid levels [62]. Interestingly, further analysis of nutrient composition revealed that participants with dyslipidemia scored lower on energy, protein, carbohydrate, total fat, saturated fat, and selenium levels than those without it. It is presumed that individuals with dyslipidemia have already begun to undergo dietary changes after the diagnosis. Another NHANES study reported that participants with chronic diseases such as dyslipidemia, DM, or hypertension paid more attention to the nutritional labels of foods. These individuals occasionally exhibit inappropriate dietary patterns [63]. However, participants with dyslipidemia still had higher overall DII scores than those without it. Diet should be considered as a whole, and participants should be more concerned about diet patterns than restricting or supplementing the intake of a single nutrient [64]. Research has shown that a single dietary supplement tends toward negative results in reducing mortality from CVDs and other causes [65]. Participants with CVDs are more likely to benefit from a healthy dietary pattern than from a single dietary supplement. Another efficient strategy is to implement nutritional content ratings on food packaging beyond the mere listing of calories and ingredients to comprehensively evaluate nutrition and mitigate the risk of CVDs [66].

The subgroup analysis results showed a stronger association between DII and dyslipidemia in participants with an annual family income <$20,000. It is consistent with the global trend of dyslipidemia prevalence shifting from high-income to low-income countries [67, 68]. Subgroup analysis also showed a stronger association between DII and dyslipidemia in participants without comorbidities (CHF, DM, stroke, CAD, cancer) compared to those with them. Similarly, different BMI categories did not affect the association between DII and dyslipidemia (*P*_for interaction_ = 0.34), but for participants with a BMI of 25–30 kg/m^2^, an increase in DII was associated with an increase in dyslipidemia (*P*_for trend_ = 0.02). Possible causes are associated with the comorbid dyslipidemia that participants with aforementioned chronic diseases or overweight individuals (BMI > 25 kg/m^2^) might have already had, thus weakening the association between DII and dyslipidemia. These results suggested that special attention should be given to the intake of anti-inflammatory diet, for individuals with fewer comorbidities, a BMI of 20–25 kg/m^2^, and an annual family income <$20,000, as their dietary intake had a stronger association with dyslipidemia.

## Limitations

There were unavoidable limitations; therefore, the conclusions of this study should be interpreted with caution. First, as this was a cross-sectional study, a causal explanation for the relationship between DII and dyslipidemia cannot be provided. Second, data from the NHANES database were subject to memory bias and random errors. This study did not include individuals below the age of 20 years or those without serum sample, which may have resulted in data bias. Furthermore, as a result of the database’s design, some individual information among the participants could not be extracted, potentially introducing bias, such as energy intake restriction and inflammatory-related diseases. Recently, studies have reported the Energy-adjusted Dietary Inflammatory Index (E-DII) [69, 70] that aims to mitigate the bias resulting from differences in energy intake. In future research, we plan to incorporate E-DII and further validate its effectiveness in addressing this source of bias.

## Conclusions

The findings of our study indicates a close association between DII and dyslipidemia, as evidenced by higher scores on certain proinflammatory diets among participants with dyslipidemia compared to those without it. Moreover, our study indicates that a pro-inflammatory diet may play a role in unfavorable consequences and is linked to both all-cause mortality and cardiovascular death in patients with dyslipidemia.

### Electronic supplementary material

Below is the link to the electronic supplementary material.

Supplementary Material 1

## List of abbreviations

AMPM: Automated Multiple Pass methods
BMI: Body Mass Index
CAD: Coronary Artery Disease
CHF: Congestive Heart Failure
CIs: Confidence Intervals
CRP: C reactive protein
CVDs: Cardiovascular Diseases
DBP: Diastolic Blood Pressure
DII: Dietary Inflammatory Index
DM: Diabetes Mellitus
HDL: High-Density Lipoprotein
HRs: Hazard ratios
LDL: Low-Density Lipoprotein
MUFA: Monounsaturated Fatty Acids
NHANES: National Health and Nutrition Examination Survey
OR: Odds Ratio
PUFA: Polyunsaturated Fatty Acids
SBP: Systolic Blood Pressure
TC: Total Cholesterol
TG: Triglycerides
