# Supplementary material for: Dietary inflammation index is associated with dyslipidemia: evidence from national health and nutrition examination survey, 1999–2019
Source: Lipids Health Dis. 2023 Sep 9;22:149. doi: 10.1186/s12944-023-01914-z (PMC10492364; doi:10.1186/s12944-023-01914-z)
Supplement: Supplementary file 1 — Supplementary Material 1 [file 12944_2023_1914_MOESM1_ESM.docx]

**Supplementary File**

Table S1 Baseline characteristics of participants (n = 17 820) across quartiles of DII

| Variable | Q1(-4.67,0.26] | Q2(0.26,1.76] | Q3(1.76,2.96] | Q4(2.96,5.50] | *P* value |
| --- | --- | --- | --- | --- | --- |
|  | N=4552^a^ | N=4556^a^ | N=4551^a^ | N=4549^a^ |  |
| Age | 47.57 ± 0.41 | 48.19 ± 0.38 | 47.50 ± 0.40 | 46.58 ± 0.41 | 0.01 |
| Sex (%) |  |  |  |  | < 0.01 |
| Female | 1634(36.51) | 2046(46.94) | 2404(57.87) | 2796(64.80) |  |
| Male | 2820(63.49) | 2414(53.06) | 2046(42.13) | 1660(35.20) |  |
| Race (%) |  |  |  |  | < 0.01 |
| Mexican American | 826(7.38) | 797(7.53) | 768(6.60) | 690(6.83) |  |
| Non-Hispanic Black | 642(6.93) | 780(8.76) | 975(11.95) | 1062(12.93) |  |
| Non-Hispanic White | 2246(76.25) | 2144(72.52) | 2045(71.69) | 2028(69.45) |  |
| Other Hispanic | 316(4.05) | 360(5.33) | 357(5.16) | 387(5.71) |  |
| Other Race | 424(5.39) | 379(5.86) | 305(4.61) | 289(5.07) |  |
| Annual family income (%) |  |  |  |  | < 0.01 |
| <$20,000 | 884(15.29) | 1047(17.40) | 1184(22.67) | 1386(25.87) |  |
| ≥$20,000 | 3570(84.71) | 3413(82.60) | 3266(77.33) | 3070(74.13) |  |
| Education (%) |  |  |  |  | < 0.01 |
| < High school | 382(3.81) | 471(5.40) | 515(6.52) | 587(7.45) |  |
| > High school | 2700(68.02) | 2443(61.31) | 2177(54.77) | 1858(45.71) |  |
| High school | 1372(28.17) | 1546(33.30) | 1758(38.71) | 2011(46.85) |  |
| Fast triglyceride (mmol/L) | 1.37 ± 0.02 | 1.43 ± 0.02 | 1.43 ± 0.02 | 1.41 ± 0.02 | 0.06 |
| Fast total cholesterol (mmol/L) | 5.02 ± 0.03 | 5.11 ± 0.02 | 5.13 ± 0.03 | 5.08 ± 0.03 | 0.01 |
| HDL cholesterol (mmol/L) | 1.38 ± 0.01 | 1.38 ± 0.01 | 1.38 ± 0.01 | 1.36 ± 0.01 | 0.11 |
| LDL cholesterol (mmol/L) | 3.00 ± 0.03 | 3.08 ± 0.02 | 3.09 ± 0.02 | 3.08 ± 0.02 | 0.07 |
| CRP (mg/L) | 0.33 ± 0.02 | 0.42 ± 0.02 | 0.41 ± 0.02 | 0.52 ± 0.03 | < 0.01 |
| BMI (kg.m2) | 27.68 ± 0.14 | 28.25 ± 0.18 | 28.64 ± 0.14 | 29.10 ± 0.16 | < 0.01 |
| SBP (mmHg) | 121.15 ± 0.38 | 122.37 ± 0.42 | 122.35 ± 0.39 | 122.05 ± 0.41 | 0.08 |
| DBP (mmHg) | 71.93 ± 0.31 | 71.01 ± 0.26 | 70.96 ± 0.36 | 70.25 ± 0.33 | < 0.01 |
| Dyslipidemia (%) | 3091(69.96) | 3249(73.43) | 3284(74.62) | 3357(76.26) | < 0.01 |
| Non- Dyslipidemia (%) | 1363(30.04) | 1211(26.57) | 1166(25.38) | 1099(23.74) | < 0.01 |
| Hypertension (%) | 1748(35.14) | 1870(37.92) | 1907(37.17) | 2043(40.39) | 0.02 |
| DM (%) | 1384(22.39) | 1477(25.63) | 1476(24.40) | 1638(28.70) | < 0.01 |
| CHD (%) | 178(3.73) | 194(3.85) | 169(3.27) | 194(3.47) | 0.69 |
| CHF (%) | 89(1.51) | 130(2.64) | 137(2.53) | 172(2.66) | 0.04 |
| Stroke (%) | 107(1.90) | 155(2.95) | 158(2.51) | 220(3.44) | < 0.01 |
| Cancer (%) | 448(9.26) | 405(9.61) | 361(9.10) | 432(9.52) | 0.94 |
| Smoke (%) |  |  |  |  | < 0.01 |
| Former | 1281(30.50) | 1232(28.28) | 1085(24.48) | 981(20.83) |  |
| Never | 2435(54.27) | 2409(51.13) | 2373(51.37) | 2290(49.36) |  |
| Now | 738(15.23) | 819(20.59) | 992(24.14) | 1185(29.81) |  |
| Alcohol (%) |  |  |  |  | < 0.01 |
| Former | 620(12.32) | 696(13.59) | 838(16.39) | 950(18.70) |  |
| Heavy | 900(18.70) | 886(19.67) | 890(20.68) | 855(21.17) |  |
| Mild | 1820(44.66) | 1636(38.71) | 1422(34.38) | 1253(29.49) |  |
| Moderate | 646(16.10) | 679(17.50) | 701(16.72) | 647(17.15) |  |
| Never | 468(8.22) | 563(10.53) | 599(11.84) | 751(13.49) |  |

Note: Mean ± SE for continuous variables; *P* value was calculated by weighted t test, Number (%) for categorical variables: The *P* value was calculated by weighted chi-square test. ^a^Unweighted number of observations in dataset.

Table S2 Comparison of components of DII by baseline

| Variable | Total | Non-hyperlipidemia | Hyperlipidemia | *P* value |
| --- | --- | --- | --- | --- |
| Energy | -0.003 ± 0.002 | 0.017 ± 0.003 | -0.010 ± 0.002 | < 0.01 |
| Protein | -0.001 ± 0.000 | 0.000 ± 0.000 | -0.002 ± 0.000 | < 0.01 |
| Carbohydrates | -0.018 ± 0.001 | -0.009 ± 0.002 | -0.021 ± 0.001 | < 0.01 |
| Fiber | 0.199 ± 0.008 | 0.158 ± 0.011 | 0.213 ± 0.009 | < 0.01 |
| Total fat | 0.029 ± 0.003 | 0.051 ± 0.006 | 0.020 ± 0.003 | < 0.01 |
| Saturated fat | -0.069 ± 0.004 | -0.042 ± 0.007 | -0.079 ± 0.004 | < 0.01 |
| MUFA | 0.000 ± 0.000 | -0.001 ± 0.000 | 0.000 ± 0.000 | < 0.01 |
| PUFA | -0.055 ± 0.003 | -0.080 ± 0.006 | -0.046 ± 0.004 | < 0.01 |
| Cholesterol | -0.023 ± 0.001 | -0.021 ± 0.002 | -0.024 ± 0.001 | 0.14 |
| Vitamin A | 0.188 ± 0.003 | 0.180 ± 0.004 | 0.190 ± 0.003 | 0.04 |
| b carotene | 0.342 ± 0.006 | 0.333 ± 0.009 | 0.345 ± 0.006 | 0.21 |
| thiamin | 0.014 ± 0.001 | 0.008 ± 0.002 | 0.016 ± 0.001 | < 0.01 |
| riboflavin | -0.013 ± 0.001 | -0.016 ± 0.001 | -0.012 ± 0.001 | < 0.01 |
| niacin | 0.031 ± 0.002 | 0.019 ± 0.003 | 0.036 ± 0.002 | < 0.01 |
| Vitamin b6 | -0.078 ± 0.003 | -0.099 ± 0.005 | -0.071 ± 0.004 | < 0.01 |
| Folic acid | 0.105 ± 0.002 | 0.100 ± 0.003 | 0.107 ± 0.002 | 0.02 |
| vb12 | -0.019 ± 0.001 | -0.017 ± 0.002 | -0.019 ± 0.001 | 0.21 |
| Vitamin C | 0.188 ± 0.005 | 0.180 ± 0.007 | 0.191 ± 0.006 | 0.18 |
| Vitamin D | 0.211 ± 0.005 | 0.206 ± 0.008 | 0.213 ± 0.006 | 0.41 |
| Vitamin E | 0.141 ± 0.004 | 0.108 ± 0.007 | 0.153 ± 0.005 | < 0.01 |
| Mg | 0.055 ± 0.004 | 0.026 ± 0.006 | 0.066 ± 0.005 | < 0.01 |
| Fe | 0.001 ± 0.000 | 0.003 ± 0.001 | 0.000 ± 0.000 | < 0.01 |
| Zinc | -0.020 ± 0.003 | -0.036 ± 0.006 | -0.015 ± 0.004 | 0.01 |
| Selenium | -0.096 ± 0.001 | -0.104 ± 0.003 | -0.093 ± 0.001 | < 0.01 |
| Caffeine | 0.084 ± 0.000 | 0.084 ± 0.000 | 0.084 ± 0.000 | 0.01 |
| Alcohol | 0.162 ± 0.004 | 0.140 ± 0.006 | 0.169 ± 0.004 | < 0.01 |
| N3 fatty acids | 0.267 ± 0.001 | 0.268 ± 0.002 | 0.266 ± 0.001 | 0.50 |
| N6 fatty acids | -0.056 ± 0.001 | -0.065 ± 0.002 | -0.053 ± 0.001 | < 0.01 |
| DII index | 1.368 ± 0.030 | 1.225 ± 0.043 | 1.420 ± 0.033 | < 0.01 |

Note: Mean ± SE for continuous variables; *P* value was calculated by weighted t test
